# External Validation of the Martini‐Klinik Nomogram Predicting the Indication for Adjuvant Radiation Therapy According to EAU Guidelines Among Patients With High‐Risk Prostate Cancer After Radical Prostatectomy

**DOI:** 10.1002/pros.70135

**Published:** 2026-01-29

**Authors:** Carolin Siech, Tonio Brüggemann, Quynh Chi Le, Benedikt Lauer, Clara Humke, Felix Preisser, Tobias Maurer, Maximilian Kriegmair, Markus Graefen, Thomas Steuber, Mike Wenzel, Felix K. H. Chun, Philipp Mandel

**Affiliations:** ^1^ Department of Urology Goethe University Frankfurt, University Hospital Frankfurt am Main Germany; ^2^ Martini‐Klinik Prostate Cancer Center University Hospital Hamburg‐Eppendorf Hamburg Germany; ^3^ Department of Urology University Hospital Hamburg‐Eppendorf Hamburg Germany; ^4^ Department of Urology University Medical Center Mannheim, University of Heidelberg Heidelberg Germany; ^5^ Department of Urology Planegg Planegg Germany

**Keywords:** adjuvant radiation therapy, D'amico risk groups, nomogram, prostate cancer, radical prostatectomy

## Abstract

**Objective:**

To externally validate the Martini‐Klinik nomogram based on patient and clinical tumor characteristics predicting the indication for adjuvant radiation therapy after radical prostatectomy according to guideline recommendations of the European Association of Urology (EAU) in high‐risk prostate cancer patients treated with radical prostatectomy.

**Methods:**

Relying on a tertiary‐care database, we identified high‐risk prostate cancer patients treated with radical prostatectomy (01/2014‐12/2024). External validation was assessed in terms of accuracy, calibration and net benefit, using decision curve analyses.

**Results:**

Of 404 high‐risk prostate cancer patients, 182 (45%) had the indication for adjuvant radiation therapy according to current EAU guidelines. The nomogram predicted the outcome with 79% accuracy. A high level of agreement between the predicted and observed probability of indication for adjuvant radiation therapy was observed. Minimal overestimation from the ideal predictions were noted for predicted probabilities between 25% and 50%, as well as a minimal underestimation between the predicted probability of 0% and 20% as well as 75% and 100%. In decision curve analyses, the use of the nomogram resulted in greater net benefit for all threshold probabilities between 25% and 95%, relative to both competing strategies – none or all treated.

**Conclusions:**

This external validation within a contemporary tertiary‐care cohort confirmed the ability of the Martini‐Klinik nomogram to predict the indication for adjuvant radiation therapy after radical prostatectomy according to current EAU guideline recommendations in high‐risk prostate cancer patients. The present nomogram may support clinicians in preoperative patient counseling about the risk for adjuvant treatment.

## 1 Introduction

Adjuvant treatments following radical prostatectomy (RP), such as radiation therapy, are recommended for selected prostate cancer patients at high risk of disease recurrence after surgery [1, 2, 3, 4, 5, 6]. Recent guidelines of the European Association of Urology (EAU) recommend adjuvant radiation therapy for patients with pathological negative lymph nodes (pN0) with Gleason Grade Group 4 or 5 and non‐organ confined pathological tumor stage (pT3) with or without positive surgical margins, and in cases of pathological lymph‐node invasion (pN1) [5, 6]. These patients with adverse pathology have been shown to benefit from adjuvant radiation therapy [3, 4]. The potential benefits of adjuvant radiation therapy must be carefully weighed against the risks of adverse effects, within the framework of shared decision‐making. Side effects of adjuvant radiation therapy include increased rates of genitourinary toxicity (e.g. urinary incontinence, urethral stricture), gastrointestinal toxicity (e.g. proctitis, rectal bleeding), and erectile dysfunction [2]. Moreover, the need for adjuvant radiation therapy may potentially lead to regret regarding the initial therapeutic choice for RP [7, 8, 9]. Shakespeare et al. demonstrated a fourfold higher rate of decisional regret among patients who were advised to undergo adjuvant therapy postoperatively, compared to those who were treated with primary radiation therapy [9]. This difference may be attributed to the preoperative uncertainty surrounding the necessity of adjuvant treatment. Although all patients are preoperatively informed about the potential risk of adjuvant therapies, it may be difficult to quantify the risk. Therefore, in the context of preoperative patient counseling, it may be valuable to rely on a predictive model that accurately estimates the risk of adjuvant radiation therapy based on variables known prior to definitive treatment decision‐making. Recently, our working group developed a nomogram for the preoperative prediction of adjuvant radiation therapy following RP, in accordance with current EAU guidelines [10]. Relying on a cohort of 5691 patients with high‐risk prostate cancer treated at a high‐volume tertiary‐care center, namely Martini‐Klinik (Hamburg, Germany), the new model is based on preoperatively known patient and clinical characteristics, namely prostate volume, prostate‐specific antigen (PSA)‐value, clinical tumor stage (cTstage), number of sampled biopsy cores, proportion of positive cores in biopsy, and Gleason Grade Group in biopsy to predict the indication for adjuvant radiation therapy based on EAU guideline recommendations [10]. However, this novel nomogram has not yet undergone external validation, which represents an important frequently overlooked step in the development of multivariable models before clinical implementation can be considered [11]. Therefore, the present analyses evaluated the performance in terms of accuracy, calibration, and net benefit of the Martini‐Klinik nomogram in a contemporary external European tertiary‐care center cohort.

## 2 Materials and Methods

### 2.1 Study Population

**Figure 1:**
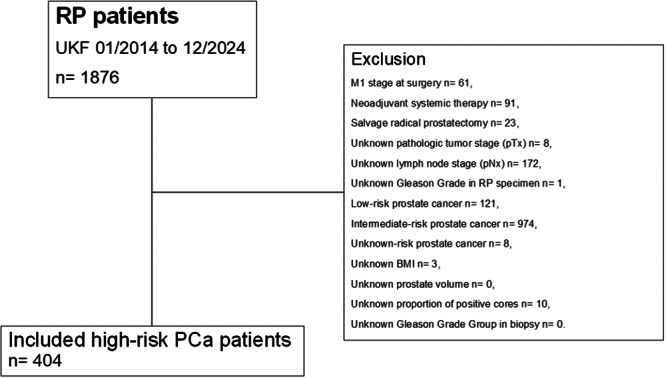
Consort diagram. BMI = body mass index, PCa = prostate cancer, pN = pathologic lymph node stage at surgery, pT = pathologic tumor stage at surgery, RP = radical prostatectomy, UKF = University hospital Frankfurt.

Relying on our prospectively maintained institutional tertiary‐care prostate cancer database, D'Amico high‐risk prostate cancer patients who were treated with RP at the Department of Urology of the Goethe University Hospital Frankfurt (Frankfurt am Main, Germany) between January 2014 and December 2024 were retrospectively identified (Figure 1). Following the previous methodology of the initially published Martini‐Klinik nomogram by Siech et al., all patients with clinical suspicion of metastases at time of surgery (cM1, *n* = 61), neoadjuvant systemic therapy (chemotherapy and/or hormonal therapy, *n* = 91), and previous radiation therapy of the prostate (salvage radical prostatectomy, *n* =23) were excluded [10]. A complete case analysis was performed. Hence, patients with incomplete data regarding pathological tumor (pT, *n* = 8) and lymph node stage (pN, *n* = 172), Gleason Grade Group in biopsy (*n* = 0) and RP specimen (*n*= 1), body mass‐index (BMI, *n* = 3), prostate volume (*n* = 0), and proportion of positive biopsy cores (*n* = 10) were excluded from the study cohort. Importantly, exclusion criteria were identical to those used in the original Martini‐Klinik nomogram to ensure methodological consistency.

Approval by the local ethics committee was obtained prior to data collection. All patients gave their written informed consent for data collection. Reporting followed the precepts established by the Helsinki Declaration.

### 2.2 Covariates and Study Endpoints

The primary endpoint of the previously designed nomogram (relying on 5691 high‐risk prostate cancer patients treated with RP at the Martini‐Klinik (Hamburg, Germany) between 01/2010 and 08/2024) represented the prediction of the indication for adjuvant radiation therapy after RP according to current guideline recommendations of the EAU [5, 6, 10]. The included covariates that represented significant predictors of the indication for adjuvant radiation therapy after RP were as follows: prostate volume (> 45 vs. 25–45 *vs.* < 25 cm^3^), PSA ‐value (> 20 vs. 10–20 vs. < 10 ng/ml), clinical tumor stage based on digital rectal examination (cT‐stage: cTx vs. cT3/cT4 vs. cT2 vs. cT1), number of positive cores at biopsy (> 12 vs. ≤ 12), proportion of positive cores in biopsy (continuously, in %), highest Gleason Grade Group per core in biopsy (5 vs. 4 vs. 3 vs. 2 vs. 1) [10].

### 2.3 Statistical Analyses

Four analytical steps were completed. First, patient, clinical, and pathological characteristics were tabulated. Descriptive statistics were presented using frequency and proportions for categorical variables and median with interquartile range (IQR) for continuously coded variables. The non‐parametric Wilcoxon rank sum test evaluated the statistical significance of medians' differences for continuous variables. Pearson's Chi‐squared test examined the statistical significance in proportions' differences for categorical variables. External validation was derived from the initial odds ratios (ORs) and intercepts of the above‐mentioned covariates from the novel multivariable logistic regression model from the Martini‐Klinik presented by Siech et al. [10]. As recommended by the transparent reporting of a multivariable prediction model for individual prognosis or diagnosis (TRIPOD) statement, external validation of the predictive model was evaluated in terms of discrimination, calibration, and net benefit [12]. Discrimination was quantified using the Harrell's concordance index (c‐index) [13]. Moreover, the area under the curve (AUC) of the receiver operating characteristic (ROC) curve was plotted and 95% confidence interval (CI) were estimated after 2000 bootstrap resamples [11, 13]. Calibration was assessed using calibration‐in‐the‐large (CILR) and calibration slope. The extent of over‐ and underestimation between the observed probability of indication for adjuvant radiation therapy and predicted probability for the new Martini‐Klinik nomogram performance in the external validation cohort was graphically described using calibration plots. A decision‐curve‐analysis (DCA) was used to evaluate the net benefit associated with the use of the new Martini‐Klinik multivariable model in the external validation cohort, relative to random consideration of all patients or no patients for adjuvant radiation therapy after RP.

Statistical significance level was set at *p* < 0.05. R software environment was used for statistical computing and graphics (R version 4.3.2; R Foundation for Statistical Computing, Vienna, Austria) [14].

## 3 Results

### 3.1 Patient and Clinical Characteristics

Overall, 404 D'Amico high‐risk prostate cancer patients were identified and deemed eligible for study inclusion (Figure 1). Median age at surgery was 67 years (IQR 62–72 years), median BMI was 26.7 kg/m^2^ (IQR 24.5–29.3 kg/m^2^), median prostate volume was 44 cm^3^ (IQR 31–55 cm^3^), and median preoperative PSA‐value was 11.2 ng/mL (IQR 6.5–25.0 ng/mL; Table 1). Addressing clinical tumor characteristics, most patients exhibited cT2 stage (54%), and harbored Gleason Grade Group 4 in biopsy (43%). Median proportion of positive cores in biopsy was 46% (IQR 29–64%). 45% of overall included patients had ≤ 12 sampled biopsy cores.

**Table 1:** Patient and clinical characteristics of 404 D'Amico high‐risk prostate cancer patients treated with radical prostatectomy between 01/2014 and 12/2024 at the University Hospital Frankfurt.

Characteristic		Overall, *n* = 404	Indication for adjuvant radiation therapy, *n* = 182 (45%)^a^	No indication for adjuvant radiation therapy, *n* = 222 (55%)^a^	*p*‐value^b^
Age at surgery (in years)		67 (62, 72)	68 (63, 72)	67 (62, 72)	0.2
BMI (in kg/m^2^)		26.7 (24.5, 29.3)	26.7 (24.4, 29.3)	26.7 (24.7, 29.4)	0.8
Prostate volume (in cm^3^)		44 (31, 55)	45 (34, 60)	40 (30, 55)	0.1
	< 25	28 (7%)	8 (5%)	20 (9%)	0.1
	25–45	202 (50%)	88 (48%)	114 (51%)	
	> 45	174 (43%)	86 (47%)	88 (40%)	
PSA (in ng/mL)		11.2 (6.5, 25.0)	12.7 (7.2, 25.8)	10.0 (6.1, 24.9)	**0.016**
	< 10	174 (43%)	64 (35%)	110 (50%)	**< 0.001**
	10–20	92 (23%)	56 (31%)	36 (16%)	
	> 20	138 (34%)	62 (34%)	76 (34%)	
cTstage	cT1	145 (36%)	41 (23%)	104 (47%)	**< 0.001**
	cT2	217 (54%)	110 (60%)	107 (48%)	
	cT3/cT4	42 (10%)	31 (17%)	11 (5%)	
Number of sampled biopsy cores ≤ 12		180 (45%)	90 (49%)	90 (41%)	0.1
Proportion of positive cores in biopsy (in %)		46 (29, 64)	55 (35, 75)	38 (24, 54)	**< 0.001**
Gleason Grade Group in biopsy	1	15 (3%)	2 (1%)	13 (6%)	**< 0.001**
	2	44 (11%)	10 (5%)	34 (15%)	
	3	43 (11%)	21 (12%)	22 (10%)	
	4	172 (43%)	60 (33%)	112 (51%)	
	5	130 (32%)	89 (49%)	41 (18%)	


^a^ Median (interquartile range); *n* (%).

^b^ Wilcoxon rank sum test; Pearson's Chi‐square test.

### 3.2 Pathological Characteristics

**Figure 2:**
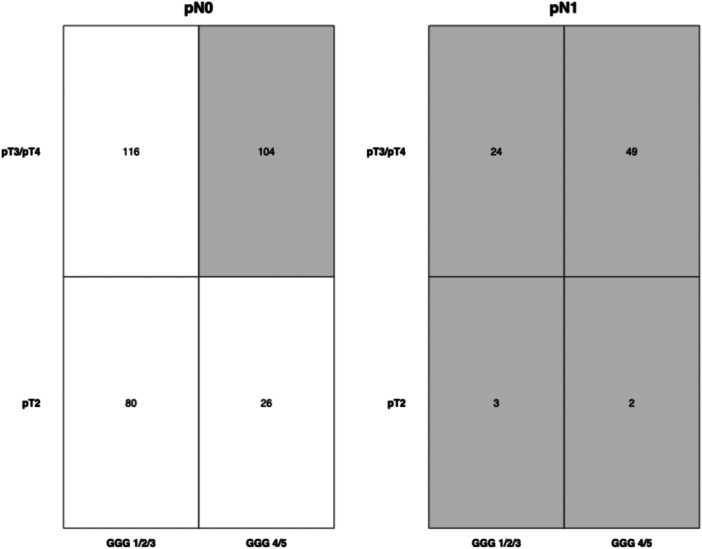
Heat map depicting the distribution of pathologic tumor stage and Gleason Grade Group according to pathologic lymph node stage in radical prostatectomy specimen in 404 high‐risk prostate cancer patients. Gray overlay highlights patients with indication for adjuvant therapy after radical prostatectomy according to the current prostate cancer guidelines of the European Association of Urology (EAU 2025). GGG = Gleason Grade Group at surgery, pN = pathologic lymph node stage at surgery, pT = pathologic tumor stage at surgery.

Among 404 high‐risk prostate cancer patients treated with RP, 182 (45%) had indication for adjuvant radiation therapy (Figure 2). Of these, 78 patients (43%) had pathological positive lymph nodes (pN1), 177 (97%) had a non‐organ confined tumor stage (pT3/pT4), 155 (85%) harbored Gleason Grade Group 4 or 5, and 119 (65%) had positive surgical margins (R1) in RP specimen.

### 3.3 External Validation of the Martini‐Klinik Nomogram Predicting Indication for Adjuvant Radiation Therapy in High‐Risk Prostate Cancer Patients After Radical Prostatectomy

**Figure 3:**
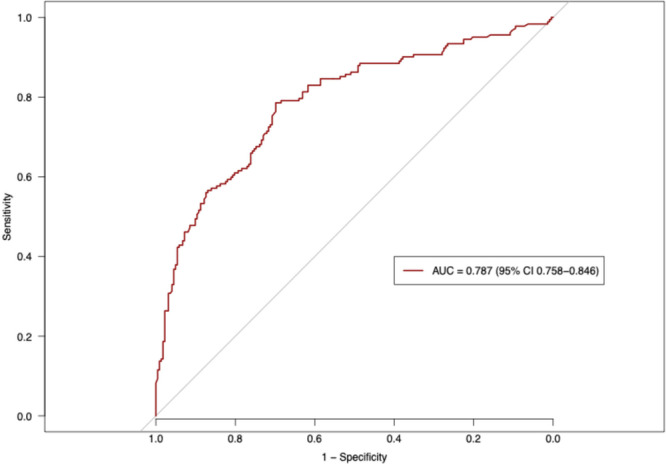
Area under the curve (AUC) and 95% confidence interval estimated after 2000 bootstrap resamples on receiver operating characteristic (ROC) curve used to predict indication for adjuvant radiation therapy in the independent external validation cohort of 404 high‐risk prostate cancer patients after radical prostatectomy. [Color figure can be viewed at wileyonlinelibrary.com]

**Figure 4:**
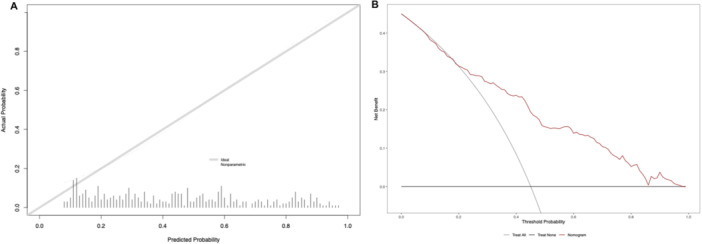
(A) Calibration plot illustrating the relationship between the observed probability of the indication for adjuvant radiation therapy and predicted probability for the new Martini‐Klinik nomogram performance in the external validation cohort. The ideal relationship between the predicted and observed probability is depicted by the gray line (Area below the gray line= where the model's risk estimates are too high; area above the gray line= where the model's risk estimates are too low) and (B) Decision‐curve analysis depicting the net benefit of the new Martini‐Klinik nomogram in the external validation cohort (red line), relative to random consideration of all patients for adjuvant radiation therapy (grey line) and consideration of no patients for adjuvant radiation therapy after radical prostatectomy (black line). [Color figure can be viewed at wileyonlinelibrary.com]

The published and now externally validated nomogram based on preoperative patient and clinical tumor characteristics predicted the indication for adjuvant radiation therapy after RP according to current EAU guideline recommendations. In our external high‐volume tertiary‐care RP cohort the accuracy was 78.7% (AUC= 0.787; 95% CI 0.758–0.846; Figure 3). On the calibration plot, a high agreement between the predicted and observed probabilities for the indication for adjuvant radiation therapy was observed (Figure 4A). Minimal deviation from the ideal predictions were noted for predicted probabilities between 25% and 50%, with an overestimated probability of indication for adjuvant radiation therapy in that range, as well as a minimal underestimation between the predicted probability ranges of 0% and 20% as well as between 75% and 100%. In decision curve analyses, the use of the new Martini‐Klinik nomogram resulted in a greater net benefit for all threshold probabilities between 25% and 95%, relative to both competing strategies, namely random consideration of all high‐risk prostate cancer patients for adjuvant radiation therapy and random consideration of no patients for adjuvant radiation therapy (Figure 4B).

## 4 Discussion

The estimated risk of requiring adjuvant radiation therapy after RP may influence the choice of initial therapeutic strategy and potentially affect therapeutic decision regret in prostate cancer patients [7, 8, 9]. A nomogram capable of quantifying this risk for need of adjuvant radiation therapy may serve as a valuable tool in the context of shared decision‐making prior to primary treatment, helping to reduce post‐treatment decision regret. Moreover, such a nomogram may provide both urologists and radiation oncologists with significant support during patient counseling regarding curative treatment options for non‐metastatic high‐risk prostate cancer. It is in this context, a new Martini‐Klinik nomogram was recently developed [10]. In the present study, we completed the first external validation of the new Martini‐Klinik nomogram predicting the indication for adjuvant radiation therapy in high‐risk prostate cancer patients after RP in accordance with current EAU guideline recommendations [5, 6, 10]. Data from 404 D'Amico high‐risk prostate cancer patients treated with RP between January 2014 and December 2024 were included in the present analyses. External validation was assessed in terms of discrimination, calibration and clinical net benefit and we made several noteworthy observations.

First, in our independent external validation cohort, 45% of high‐risk prostate cancer patients met the criteria for adjuvant radiation therapy according to current EAU guidelines. This rate is higher than the one observed in the original Martini‐Klinik cohort, in which 38% of patients had an indication for adjuvant radiation therapy after RP [10]. This discrepancy may be attributed to a difference in clinical characteristics between the two study cohorts. Specifically, in our study cohort patients were more frequently classified as cT3/cT4 compared to the Martini‐Klinik cohort (10 vs. 4%). Additional differences were observed for median prostate volume (44 vs. 32 cm^3^) and in the proportion of patients who had ≤ 12 sampled biopsy cores (45 vs. 77%) [10]. Second, these differences in patient and clinical characteristics translated into differences in pathological characteristics between the two study cohorts. In the present external validation cohort compared to the initial Martini‐Klinik study cohort, the proportions of patients with non‐organ confined tumor stage (pT3/pT4: 73 vs. 65%) and Gleason Grade Group 4 or 5 (45 vs. 25%) were significantly higher, leading to a higher proportion of patients with an indication for adjuvant radiation therapy observed in our cohort [10]. Differences between the present cohort and the original Martini‐Klinik cohort may be explained by referral patterns. Martini‐Klinik (Hamburg, Germany) represents the largest prostate cancer center in Germany and receives national referrals, including patients who might have earlier access to early detection programs. Conversely, patients treated at the University Hospital Frankfurt are predominantly referred from the surrounding region (Hesse, Northern Bavaria, Rhineland‐Palatinate, and Saarland). This difference in referral patterns may contribute to the higher prevalence of advanced pathological features observed in the present study cohort.

Third, external validation of the Martini‐Klinik nomogram yielded an accuracy of 79% in predicting indications for adjuvant radiation therapy according to current EAU guidelines. This observation surpasses the accuracy reported in the initial presentation of the new nomogram by Siech et al. [10], where a c‐index of 0.761 (95% CI 0.749–0.776) was achieved after 2000 bootstrap resamples for internal validation. The c‐index observed in our external validation exceeds the commonly accepted threshold of 0.700, which should be exceeded to warrant consideration for individualized prediction.

Fourth, the new multivariable model also demonstrated excellent calibration and discrimination and a high net benefit on DCA in the independent external validation cohort. Based on these findings, the generalizability of the new multivariable may be suggested in high‐risk prostate cancer patients treated at high‐volume tertiary‐care centers. With regards to the interpretation of the DCA, it is first necessary to define a threshold probability above which clinical action arises. In practice, this consideration corresponds to the probability threshold at which the physician will actively inform the patient of the risk of requiring adjuvant radiation therapy. The choice of threshold probability therefore depends on the preferences of both the physician and the patient. In the present DCA, the use of the new Martini‐Klinik nomogram resulted in a greater net benefit for all threshold probabilities between 25% and 95%, relative to both competing strategies, namely random consideration of all high‐risk prostate cancer patients for adjuvant radiation therapy and random consideration of no patients for adjuvant radiation therapy. For clinicians and patients whose threshold probability is greater than 25%, the use of the new multivariable model may be suggested. Conversely, for clinicians and patients whose threshold probability is below 25% there is no added value in applying the nomogram. In such cases, the “treat all” strategy, namely random consideration of all high‐risk prostate cancer patients for adjuvant radiation therapy, remains sufficient [15].

Taken together, the nomogram may be incorporated into preoperative patient counseling to assess whether radical prostatectomy alone is likely to be sufficient or whether there is a relevant risk of postoperative adjuvant radiation therapy. This information may help prepare patients who are likely to require multimodal therapy, defined as combined surgical and radiation treatment, thereby limiting undertreatment and postoperative decision regret. At the same time, the present nomogram may help avoid overtreatment by preoperatively quantifying the probability that adjuvant radiation therapy will not be indicated in a substantial proportion of high‐risk patients (55%) based on current guidelines recommendations of the EAU.

Despite its novelty and strengths, the present study has limitations. First, we relied on retrospective analyses of patients treated with RP at a single tertiary‐care center. Therefore, the present study shares the same limitations than previous retrospective single‐center studies [16, 17, 18, 19, 20, 21]. Second, as already noted in the original article by Siech et al., multiparametric magnetic resonance imaging (mpMRI) associated variables such as number, location, and degree of PIRADS lesions were not used in the development of the Martini‐Klinik nomogram [10]. Given that mpMRI is not reimbursed for early detection of prostate cancer by German public health insurance yet, mpMRI parameters were only available for a minority of patients and could therefore not be considered in the development of the multivariate model. In the mpMRI era, local staging and particularly assessment of the extracapsular extension and seminal vesicle invasion, has substantially improved [22]. Based on the observation that mpMRI‐associated variables showed improved clinical usefulness in other prediction tools, such as the Briganti 2019 nomogram for predicting lymph node involvement in prostate cancer patients [22], future iterations of the Martini‐Klinik model should examine whether mpMRI‐associated variables improve its accuracy in estimating the indication for adjuvant radiation therapy according to current EAU guidelines, as future integration of mpMRI‐derived variables may further enhance preoperative risk assessment and refine prediction of the indication for adjuvant radiation therapy. Last but not least, both study cohorts consisted of patients treated at German tertiary‐care centers. Further external validation by independent international research group is therefore warranted.

## 5 Conclusion

This external validation within a contemporary tertiary‐care cohort confirms the ability of the Martini‐Klinik nomogram to predict the indication for adjuvant radiation therapy after radical prostatectomy according to current EAU guideline recommendations in high‐risk prostate cancer patients. The nomogram may support preoperative counseling and shared decision‐making by helping to anticipate the need for multimodal therapy. Preoperative use of the novel nomogram may reduce undertreatment and postoperative decision regret, while also limiting overtreatment in patients unlikely to require adjuvant radiation therapy.

## Funding

The authors received no specific funding for this work.

## Ethics Statement

Approval by the local ethics committee has been obtained.

## Consent

The authors have nothing to report.

## Conflicts of Interest

The authors declare no conflicts of interest.

## Data Availability

All data generated or analyzed during this study are included in this article. Further enquiries can be directed to the corresponding author.
